# Biocapacity optimization in regional planning

**DOI:** 10.1038/srep41150

**Published:** 2017-01-23

**Authors:** Jianjun Guo, Dongxia Yue, Kai Li, Cang Hui

**Affiliations:** 1 Key Laboratory of Western China’s Environmental Systems (Ministry of Education), College of Earth and Environmental Sciences, Lanzhou University, Lanzhou, 730000, China; 2 Northwest Institute of Eco-Environment and Resources, Chinese Academy of Sciences, Lanzhou 730000, China; 3 Centre for Invasion Biology, Department of Mathematical Sciences, Stellenbosch University, Matieland 7602, South Africa; 4 Mathematical Biosciences Group, African Institute for Mathematical Sciences, Cape Town 7945, South Africa

## Abstract

Ecological overshoot has been accelerating across the globe. Optimizing biocapacity has become a key to resolve the overshoot of ecological demand in regional sustainable development. However, most literature has focused on reducing ecological footprint but ignores the potential of spatial optimization of biocapacity through regional planning of land use. Here we develop a spatial probability model and present four scenarios for optimizing biocapacity of a river basin in Northwest China. The potential of enhanced biocapacity and its effects on ecological overshoot and water consumption in the region were explored. Two scenarios with no restrictions on croplands and water use reduced the overshoot by 29 to 53%, and another two scenarios which do not allow croplands and water use to increase worsened the overshoot by 11 to 15%. More spatially flexible transition rules of land use led to higher magnitude of change after optimization. However, biocapacity optimization required a large amount of additional water resources, casting considerable pressure on the already water-scarce socio-ecological system. Our results highlight the potential for policy makers to manage/optimize regional land use which addresses ecological overshoot. Investigation on the feasibility of such spatial optimization complies with the forward-looking policies for sustainable development and deserves further attention.

Great efforts are needed to position the goal of sustainable development at the forefront of public attention^1^. Within the framework of sustainable development, biocapacity (BC) serves as the base for social, economic and human wellbeing^2^,^3^,^4^, which further determines the limit of human activities^5^, making the estimation of carrying capacity essential to regional planning. Many quantitative methods have been proposed for estimating biocapacity (for instance, NPP^6^, ecological footprint (EF)^7^, emergy^8^), in which the methodology of ecological footprint is of particular interest and translates the regional human demands for natural resources into the total area of different biological productive land necessary for material flow, with ecological footprint considered the demand on ecological capacity. By comparing ecological footprint with the biocapacity, policy makers can then assess the regional ecological budget, being either in surplus or an overshoot position^9^. To date, the methodology of ecological footprint has been applied to sustainable management and efficient exploitation of natural resources^10^,^11^,^12^,^13^,^14^,^15^,^16^,^17^,^18^,^19^,^20^,^21^,^22^. For instance, the biennial Living Planet Report, prepared through the cooperation of World Wildlife Fund, Zoological Society of London and Global Footprint Network, has become the magnum opus for illustrating the concept of ecological footprint and biocapacity^23^,^24^,^25^, highlighting many countries and regions across the globe being ecologically deficient. Here, we bring a deeper understanding on the management of ecological overshoot based on optimizing biocapacity.

Following its original definition by Rees and Wackernagel^26^, biocapacity can be defined as the amount of productive land and water annually available to produce resources and absorb wastes under the current management practice (Global Footprint Network, 2009). To calculate the biocapacity of a region, we first need to estimate three factors; that is, the available area of biologically productive land and water, yield factor, and equivalence factor (details of the equation see Bastianoni *et al*. and Yue *et al*.)^18^,^27^. After the conversion using the yield factor and equivalence factor, one can calculate the biocapacity with the unit of global hectare (gha). A number of methods have been proposed to improve the accuracy of estimated biocapacity. For instance, Haberl *et al*. calculated the biocapacity of Austria based on local yield for domestic extraction and global yield for imported biomass^28^. Senbel *et al*. explored the biocapacity and ecological budget of North America under several scenarios of consumption, ecological productivity and material-flow efficiency^11^. Zhao *et al*. translated energy into biological productive units and made it possible to assess resource use in ecosystems^29^. Venetoulis and Talberth changed the basis of equivalence factor to net primary productivity (NPP) to estimate carbon sequestration biocapacity^16^. Based on the emergy theory, Siche *et al*. incorporated low productivity lands into the calculation of biocapacity^30^. Yue *et al*. further demonstrated the scale effect of biocapacity, casting doubts on many regional and global studies which often rely on coarse-scale datasets^27^.

Human activities and regional planning (or policies) exert influence on many components of embedded ecosystems that provide us with essential products and services^31^. It is therefore necessary to formulate or modify the regional planning and policies for better environmental and resource management to ensure a sustainable way of harvesting ecosystem service^10^,^13^,^32^,^33^. However, the methodology of ecological footprint ignores policies and management actions, thus providing little knowledge to guiding the potential dynamics of ecological footprint and biocapacity, which influence the level of ecological overshoot^22^,^32^,^34^,^35^. The 3S technologies, including remote sensing, geographic information system and global positioning system, have come to the aid for spatially explicit assessment of biocapacity. Since Chang and Xiong and Yue *et al*. incorporated the 3S technologies into the EF methodology^13^,^36^, spatial analyses of ecological footprint and biocapacity have been increasingly emphasized in the literature^27^,^37^. However, little has been done for biocapacity optimization in a spatially explicit fashion, which could pave the way for alternative strategies for sustainable management. In addition, scenario building is a useful planning tool for constructing different models or policy measures for likely future events^38^,^39^, and are used for stakeholders to deepen their understanding of the future consequences associated with today’s human activities and decisions, against a range of possible outcomes^24^,^40^.

To this end, we provide a framework for spatially-explicit optimization of biocapacity under four scenarios and two spatial neighborhood structures. For demonstration, we choose one typical river basin in Northwest China (the Shiyang River Basin, SRB) and develop the optimal planning for regional biocapacity. With the help from numerical simulations in geographic information systems, we seek to develop a deeper understanding of biocapacity: the sensitivity of biocapacity optimization to the scenarios and neighborhood conditions, and its policy relevance to regional planning, especially water resource management. Answers to these questions enable us to identify the prospect of regional sustainable development and assist local government with viable management actions.

## Results

### Maximal biocapacity (MB) scenario

We illustrate the dynamics of different land use categories under two neighborhood conditions during the procedure of optimization in Supplementary Fig. S1, with details as insets in the figure. We calculated the transition matrix for two neighborhood conditions based on cell status pre- and post-optimization (Table 1). Evidently, maximization of biocapacity requires the transformation of barren lands into croplands, boosting up the biocapacity by nearly 3.6 × 10^5^ gha for the von Neumann neighborhood and 3.9 × 10^5^ gha for the Moore neighborhood. About 20% grazing lands need to be converted to forest lands, with a small percentage of grazing lands to croplands; the transition of grazing lands contributed an extra 2 × 10^5^ gha. Merely 1% forests were transformed into croplands, adding only 2.5 × 10^4^ gha to the regional biocapacity. No croplands were transformed into other land use types. Overall, the transformation of barren lands and grazing lands contributed the most of biocapacity increment, a rise of 13–14% biocapacity from the current stand in SRB. Moreover, larger neighborhood (Moore) brought more dramatic changes in biocapacity, regardless increasing or reducing, than smaller neighborhood (von Neumann).

### Optimal biocapacity (OB) scenario

The dynamics of different land use changes in the OB scenario under two neighborhood conditions are illustrated in Supplementary Fig. S2. Due to the less restriction for the cell status transition, the OB scenario showed wider fluctuations than the previous scenario during the simulation. The calculated transition matrices in Table 2 suggest that, again, barren lands need to be transformed into croplands so to boost up the biocapacity by 3.5–3.8 × 10^5^ gha. About 20% grazing lands were transformed into forests and croplands, increasing biocapacity by 2 × 10^5^ gha. Less than 10% forests were converted to grazing lands, 1% to croplands, together reducing biocapacity by a small amount of 0.5–1.1 × 10^4^ gha. Some croplands were converted to grazing lands and forests, reducing biocapacity by 1.9–2.0 × 10^5^ gha. Overall, a total of 3.6–3.9 × 10^5^ gha were added to the current biocapacity, representing a 7.6–8.3% increment, but lower than the increment from the MB scenario. As many cell transitions led to the decline of biocapacity, the overall biocapacity increment from the OB scenario was less than those from the MB scenario.

### Optimal biocapacity scenario with no cropland increment (OBC scenario)

The dynamics of land use change under the OBC scenario are illustrated in Supplementary Fig. S3, showing stepwise changes in some land use categories. Only 1.6–1.8% barren lands were converted to grazing lands and forests, increasing the biocapacity by merely 1.5 × 10^4^ gha (Table 3). Conversion of grazing lands to forests contributed only additional 6 × 10^4^ gha. By contrast, conversion of forests to grazing lands led to a decline of 3–3.6 × 10^4^ gha. The conversion of some croplands to forests and grazing lands led to a decline of biocapacity by 1.9–2.0 × 10^5^ gha, resulting in an overall decline of 3.1–3.4% biocapacity compared to the pre-optimization state. Compared to the OB scenario, cell transitions in the OBC were more inclined to land use categories with smaller absolute values of biocapacity due to the prohibition of cropland increment, resulting in the decline of biocapacity.

### Optimal biocapacity scenario with no water resource increment (OBW scenario)

The dynamics of land use change under the OBW scenario are presented in Supplementary Fig. S4 under two neighborhood conditions and illustrated less fluctuation compared to the OB scenario. A tiny portion of barren lands were transformed, slightly boosting up the biocapacity by 4.2–5.2 × 10^3^ gha (Table 4). Similarly, only about 2% grazing lands were converted, increasing biocapacity by nearly 10^4^ gha. Conversions of forests to grazing lands and croplands resulted in a slight decline of biocapacity by 5–5.6 × 10^3^ gha. More land use conversions happened from croplands to grazing lands and forest lands, reducing biocapacity by 1.8–2.0 × 10^5^ gha. Overall, the OBW scenario reduced the current biocapacity by 2.0 × 10^5^ gha.

### Spatial distribution of BC changes with optimization

**Figure 1:**
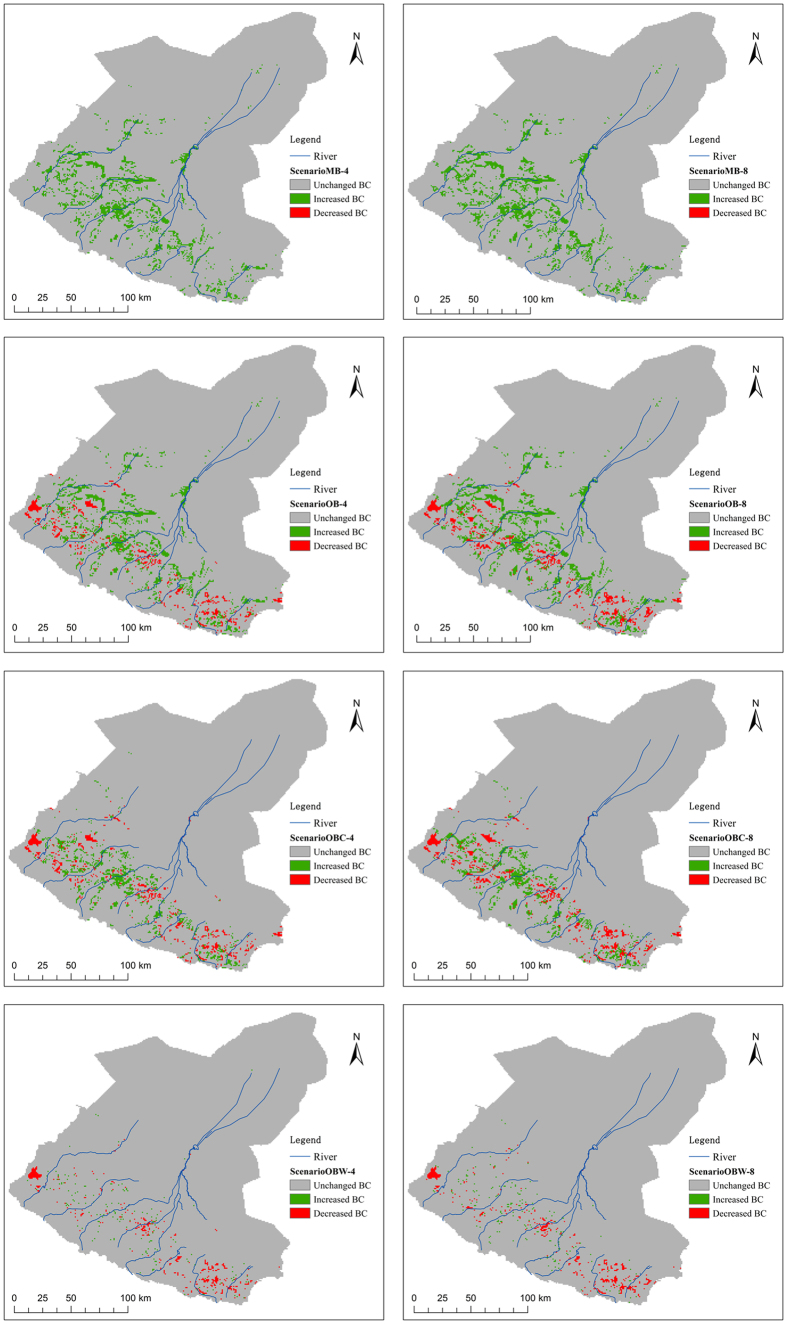
Spatial distribution of increased or decreased biocapacity under four scenarios. ScenarioMB-4 stand for the MB scenario and Von Neumann neighborhood, the others are similar. Map created using ArcMap 10.1 (http://desktop.arcgis.com/en/). *Scientific Reports* remains neutral with regard to contested jurisdictional claims in published maps.

In the MB scenario, the vast majority of regions with increased biocapacity were the upper and middle reaches of SRB (Fig. 1), while sporadic increases appeared in the lower reaches. In the OB scenario, the area with enhanced biocapacity was similar to the area from the MB scenario, while the area with reduced biocapacity mainly appeared in the upper reaches of SRB. The area with enhanced biocapacity in the OBC scenario shrank to the upper reaches of SRB, while the area with reduced biocapacity was similar to the area in the OB scenario. In the OBW scenario, the limitation of water supply sharply reduced areas that could enhance or reduce biocapacity, which are scattered across the SRB. All four scenarios indicated the upper reaches of SRB to be prioritized in the regional planning, while the lower reaches were impossible for boosting up biocapacity due to the scarcity of water resource.

### Cost of water resource

As different land use categories demand different levels of water resources, all four scenarios led to changes in the allocation and demand of water resources (Table 5). The MB scenario requires 5.16 × 10^8^ m^3^ water per annual, amounting to a 20.57% increase of the current total water use in the SRB. Water resource was required slightly less in the OB scenario, with 5% less than the demand in the MB scenario. The OBC scenario with restriction on croplands led to the minimum increase of demand on water resource (Table 5). Only the OBW scenario resulted in no changes in water demand. Transition with Moore-neighborhood led to the increase of water resource demanded for optimization.

### Changes of ecological overshoot

Changes in ecological overshoot were also calculated to assess the effectiveness of optimizing regional biocapacity (Table 6). The MB scenario led to a reduction of ecological overshoot by 47.87% (von Neumann neighborhood), followed by the OB scenario with a reduction of overshoot by 28.58%. The OBC scenario with constraints on croplands led to an increase of overshoot by 11.43% (Z = 4). Likewise, the OBW scenario with no further increase in water demand brought an increase of ecological overshoot by 13.84%.

## Discussion

Given the ongoing increase of ecological overshoot in the closed system of our planet^25^, the estimation and management of human ecological footprint (demand) relative to biocapacity (supply) becomes one of the most important issues for sustainable development^9^,^20^. The introduction of geographic information system and remote sensing paves the way for further development of biocapacity assessment^13^,^27^,^36^,^41^. In mind with reducing ecological overshoot and improving policy efficacy, we here proposed four alternative scenarios for potential boosting biocapacity through future land management policy. Given the small number of studies on biocapacity scenario analyses, the four scenarios of biocapacity optimization did not represent the full spectrum of socio-ecological complexity across the globe^9^ but only a simple list for the SRB. Different from many studies that quantify long-term future dynamics of biocapacity under the mode of ‘Business as Usual’^9^,^39^, we integrated the spatial probability model with scenarios that allow us to probe feasible biocapacity potential in a spatially explicit manner^42^. The method we developed here was based on the Cellular Automaton model (neighborhood effects) and CLUE-S model (probability distribution of various land uses) that have been used in sustainable development^43^. The developed method for biocapacity optimization can be applied to other regions where ecological overshoot can be hampered and the provision of ecosystem service assured.

As a newly developed methodology, the policy relevance of ecological footprint theory remains the subject of debate^22^,^32^,^33^,^35^,^44^. One of the potential impacts of our method is to inform the range and location of biocapacity for sustainable development. Spatially explicit results can have far-reaching policy implications for land use management on issues such as land invasion, resource allocation and regional planning. Identifying hotspots of biocapacity change or optimization can help regional prioritization and planning. Our results clearly identified the lower reach of SRB to be little prioritized in all scenarios for biocapacity optimization, suggesting the development of land use in the lower reach could have saturated; land-use management should prioritize the upper and middle reach of the basin (Fig. 1). The spatially explicit potential could guide other land use policies, e.g. financial incentives and subsidies, to encourage changes that enhance biocapacity, and to reduce the likelihood of unexpected consequences of land use change^43^,^45^.

We need to highlight that not all four scenarios are feasible in the SRB, and the feasibility of each land-use change scenario depends on the context of socio-ecological status of the region. For instance, the biocapacity of SRB increased 14% under the MB scenario and 8.34% under the OB scenario. Although croplands are critical in estimating ecological footprint and boosting biocapacity^9^,^46^, massive transition to croplands in the MB and OB scenarios could incur serious conflicts on water usage. The demand of water resource in the region could experience an increase by 20.57% under the MB scenario and 19.69% under the OB scenario. The SRB has historically been a national commodity grain base area which led to excessive consumption of water resources. Given the current water management plan (the Comprehensive Restoration Plan of the Shiyang River Basin implemented), it is impossible to foster further cropland expansion unless there are plans for inter-basin water transfer. The current planned water transfer project (the West Route of South-to-North Water Transfer Project) and less water-demanding crop choices could alleviate the current crisis of water shortage^47^. By contrast, the OBC scenario emphasizes environmental protection through the policy of Grain for Green (converting croplands to forests and grazing lands), leading to a slight reduction of biocapacity and 12.82% increase of ecological overshoot; however, the successful implementation of OBC scenario also requires a huge additional amount of water resources (Table 5). Specifically, the OBW scenario which emphasizes water conservation, brought a reduction of biocapacity and 15.17% increase of ecological overshoot, illustrating clearly the scarcity of water and signaling the current unsustainable way of development^47^. Although failing to boosting biocapacity under last two constrained scenarios, the results nonetheless provide sound assessments of the serious ecological overshoot for decision-makers. Under the premise that water resource demand cannot be satisfied through planned inter-basin water transfer, the biocapacity of the SRB is expected to shrink.

Since the theory was proposed on balancing ecological footprint with biocapacity by Rees and Wackernagel^26^, it has been applied at a variety of spatial scales, from local to global^22^,^23^,^24^,^25^. Among these studies, regional and global ecological overshoots are apparent. To this end, the potential of increasing regional biocapacity could help to curb the ever enlarging ecological overshoot. Our work highlights the potential for sustainable development through enhancing regional biocapacity. The demand of the entire humanity is currently equivalent to the service of 1.6 earths, which could reach the supply of 2.9 earths by 2050^24^. It is clear that the regenerative capacity of the earth is no longer sufficient to support our ever-increasing demands. Our approach could stimulate policy decision-making for mitigating ecological overshoot through optimizing biocapacity. Nevertheless, the gap between the demand for sustainable future of a region cannot be solely filled by the biocapacity optimization, but also on the influx of ecosystem services from other regions^9^. Indeed, unless the gap between water demand and supply can be filled by inter-basin water transfer, biocapacity optimization will have little space to maneuver. To this end, the global ecological overshoot needs a major structural change in the society^34^,^48^, either through drastically reducing current ecological footprint which is unlikely or industrial revolution to enhance/replace diminishing ecosystem services in low-valued land use.

Spatial simulation, remote sensing data and scenario analysis are powerful tools to unveil the complexity with respect to boosting biocapacity and sustainable development^39^. Following the estimation model of BC^18^,^27^, our work provides the first assessment of biocapacity optimization and policy implication in a spatially explicit framework. However, our work still has some limitations and deserves further developments. First, we simulated the neighborhood effect using the Von Neumann and Moore neighborhood, meaning that the spatial interaction of land use only happens locally^49^,^50^. Considering the autocorrelation of BC and land use^37^, there is a need to refine the knowledge for much more complex neighborhood effects in the coupling of the biocapacity optimization and regional planning. Second, we carried out the spatial optimization with a nominal resolution of 1 km. Studies have depicted the scale dependency of the GIS-based calculation of BC^27^,^37^, exploring the spatial simulation with finer scales (resolution) could help us to identify the scale effect and minimize the uncertainty about the biocapacity optimization. Moreover, we did not consider the heterogeneity of water resource demand for each land use, but only used the average amount of water used by specific land use. The use of finer resolution water use data is needed to refine biocapacity optimization and to better evaluate the true cost of water use change associated with land use transition. Third, spatial variables (i.e. environmental and socio-economic variables) have different levels of time-dependency^51^,^52^, and thus can give rise to time-dependent probability of land use transition. A more robust guideline for biocapacity optimization needs to consider the temporal dimension of land use change.

Biocapacity optimization is not only related to land use change and water resources management, but also importantly include many other management problems in regional planning; for instance, the use of financial incentives for guiding land use and development behaviors. Financial incentives which can motivate multiple stakeholders in land use and management have been well explored in the literature on the effect of alternative strategies of financial incentives on biodiversity conservation^53^, renewable energy^54^, ecosystem services and land use^55^. Local people and local governments in the SRB have been provided with multiple financial incentives and support, such as cooperative-rent, farm subsidies, mulch subsidies, natural forest protection project, degraded grassland management, to enhance sustainable development. Furthermore, in 2015, The Ecological Protection and Construction Planning in Gansu province were enforced with the goal for ensuring sustainable development and providing strong financial incentives for specific regions. However, the potential negative effects of competitive financial incentives^56^ could lead to complicated issues in regulating human behavior and financing for biocapacity optimization. The interface between biocapacity optimization and existing financial incentives can exert profound influence on land use dynamics and remains an interesting topic that deserves future attention.

## Conclusions

Biocapacity serves as the basis for human wellbeing and social-economic development^19^,^27^,^48^; it serves as an important threshold for regional sustainable development^5^,^12^,^25^,^28^. Within the EF framework, a comprehensive comparison of regional biocapacity with ecological footprint indicates the degree of sustainability^17^,^18^,^19^,^24^,^25^. A large number of studies have drawn conclusions of increasingly serious ecological overshoot in many regions^12^,^13^,^25^ and its lack of implementation in policy formulation^32^,^34^. It is therefore important to have a comprehensive understanding of the considerable potential of biocapacity along regional planning. Our simulation illustrated the magnitude of change of the ecological overshoot that can be achieved under different scenarios that represent alternative pathways of regional land and water resource management policies, shedding new light on the drivers of ecological overshoot. Although we do not provide a robust reference scenario for boosting biocapacity, we believe that the results are valuable to the “shrink and share” of ecological overshoot^9^. Putting regional planning (i.e. Grain for Green policy) or problems to be solved into spatially-explicit framework of ecological footprint theory could finally help to improve our understanding of policy efficacy and regional sustainability.

## Methods

### Study Area

**Figure 2:**
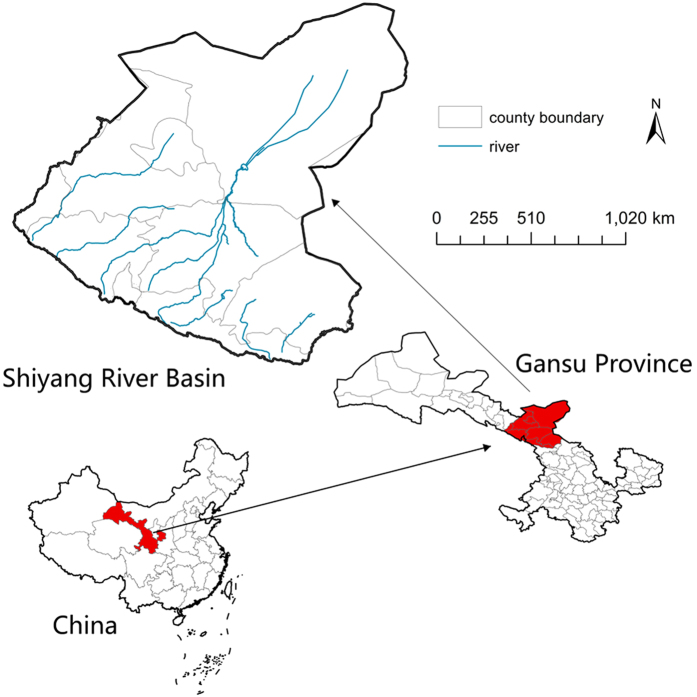
Study area of Shiyang River Basin. Map created using ArcMap 10.1 (http://desktop.arcgis.com/en/). *Scientific Reports* remains neutral with regard to contested jurisdictional claims in published maps.

The SRB is a semi-arid to arid region, with an area of 4.16 × 10^4^ km^2^ (Fig. 2), located in the transition zone of the Loess Plateau, Qinghai-Tibet Plateau and Neimenggu-Xinjiang Plateau. It is a typical mountain-oasis-desert compound ecosystem and an important barrier for ecological security in China. This area has a typical temperate continental arid climate (annual average temperature: 7.2 °C), with the mean annual precipitation from 60 mm (the northern section) to 610 mm (the southern mountainous area) but with an annual average evaporation of 2600 mm–700 mm (from north to south). The SRB faces problems of higher population density, notable conflicts between water resource supply and demand, ongoing desertification and salinization. Considering the importance of SRB to the regional ecological security in Northwestern China, research regarding environment management, sustainable development and local policies on water conservation have been prioritized. More importantly, the management of SRB has experienced challenges from increasing ecological overshoot and declining biocapacity. Biocapacity of SRB in last thirty years has fluctuated around 4 × 10^6^ gha, but the per capita biocapacity has declined in recent years by 36% due to the increase of population. In the meantime, per capita ecological footprint has drastically increased especially since 2009, with a rapidly enlarging gap between supply and demand of biocapacity^57^. This downward trend is likely to continue. In the upper reach of SRB, the biocapacity from 1985 to 2009 has showed a slight upward trend (but the average annual growth rate stays only around 0.1%) while its spatial distribution was highly scattered, together with the continuous falling of per capita biocapacity^58^. In the oasis area of the middle and lower SRB, biocapacity only increased 1.44% in the last decade (2002–2012). In particular, biocapacity in Liangzhou District (located in the middle SRB) increased only by 0.04% and that in Minqin County (located in the lower SRB) by 3.27%^59^. With such faltering growth of biocapacity accompanied by staggering increase of ecological overshoot in the SRB, spatial optimization of biocapacity becomes necessary.

### Data sources

We produced the map of biologically productive land and water at 1 km × km resolution using Landsat-TM images. Biocapacity change is a complex process driven by multiple factors, e.g. biophysical, social and economic factors. Follow the following rules of data selection: availability, quantification, spatial heterogeneity, correlation of land use change^57^,^60^, and the analysis on primary driving forces of biocapacity change^57^, we considered the following factors to affect the spatial distribution of biocapacity: GDP density per km^2^, population density per km^2^, elevation (unit: m), slope (degree), aspect (degree), and distance to the nearest main road, to the nearest water line, and to the nearest residential area. GDP and Population data were normally interpolated to prospective resolution based on data provided at the level of administrative unit^61^. The elevation, slope and aspect data were accessed from the Geospatial Data Cloud (http://www.gscloud.cn), with the original 30m-resolution, and then transformed into the 1km-resolution.

### Methods of the research

We calculated the biocapacity of SRB by the following model from Rees^7^ and Rees and Wackernagel^26^:


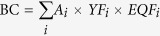


where *i* is the land category, *A*_*i*_, *YF*_*i*_, *EQF*_*i*_, are the biologically productive area, the yield factor and the equivalence factor of the *i*-th land category, respectively. Details of the model have been reviewed in many studies^18^,^27^. The order of the absolute value of biocapacity^13^ for different land use in the region is cropland >forest land >grazing land >barren land, facilitating the comparison of the contribution of these four land use types to biocapacity. Following this methodology, it is clear that land use is the most important component for calculating biocapacity.

Establishing models for simulating land use dynamics is the first step to optimize biocapacity. A variety of models and methods for simulating land use change are available, such as the Markov chain model, CLUE and CLUE-S model, Cellular Automata and so on. Markov chain models often require land use data for multiple years and are quite complicated for spatially explicit issues^62^. CLUE and CLUE-S models are based the snapshot of one-year land use data and use the suitability to indicate the spatial transfer probability of any land use; they have the advantage of producing spatially and temporally dynamic simulations^63^. However, CLUE and CLUE-S models are difficult to implement the neighborhood effect of land use change. Recently, Cellular Automata, being a dynamic simulation framework that can implement the neighborhood effect, was developed for simulating land use change^64^. Evidently, each model has its pros and cons in implementation.

Due to the strong spatial autocorrelation of land use^65^ and biocapacity^37^, the neighborhood effect should be implemented in land use change simulations. Moreover, we were limited with only one-year land use data for biocapacity optimization. Based on the independence hypothesis between suitability and neighborhood effect^66^,^67^ and the model proposed by Turner^66^, White and Engelen^68^ and Barredo *et al*.^67^, we developed a probabilistic model for optimizing biocapacity by combining the suitability of land use from the CLUE-S model and the neighborhood effect of land use based on cellular automata, with the following transition probability of different land use types:


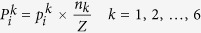


where 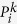 is the probability of grid *i* to become the *k*-th land use category in the future; *n*_*k*_ represents the number of the *k*-th land use category in the neighborhood of grid *i*; 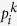 is suitability of the *k*-th land use category in grid *i* (calculated from a logistic regression^69^); Z is the number of neighbors. Similar to the cellular automaton (CA) model and CA-Markov model, we consider the classical Von Neumann neighborhood and Moore neighborhood^49^,^50^, representing different spatial effects from the nearest 4 and 8 neighboring cells.

We further proposed a list of constraints for the optimization of biocapacity: (i) setting the fishing area and built-up lands as the limited area with no transition of land use categories; (ii) the croplands cannot be changed from the desert; (iii) limiting the spread of forest in areas above the timberline^70^. We further set up four different scenarios for optimizing the biocapacity in SRB. First, in the maximal biocapacity (MB) scenario, we first calculated the probability 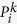 for the four land use categories in each cell and then replaced the original land use category only if the most probable land use category had a higher biocapacity than the original one. Second, in the optimal biocapacity (OB) scenario, we replaced the original land use category with the most probable land use category. Third, we implemented the OB scenario but prohibiting the increase of croplands (the OBC scenario), given the ongoing government policy on *Grain for Green*. Fourth, we carried out the OB scenario but do not allow for further increase of water demand (the OBW scenario). In each loop of the simulation, we only randomly chose one cell to update its attribute according to these four scenarios. All simulations were implemented in Matlab (MathWorks) and geographic information system (ArcGis 10.1).

## Additional Information

**How to cite this article**: Guo, J. *et al*. Biocapacity optimization in regional planning. *Sci. Rep.* **7**, 41150; doi: 10.1038/srep41150 (2017).

**Publisher's note:** Springer Nature remains neutral with regard to jurisdictional claims in published maps and institutional affiliations.

## Supplementary Material

Supplementary Information

## Figures and Tables

**Table 1:** Transformation matrix of four types’ land use according to the MB scenario (%).

Pre-\Post-opt	Barren land	Grazing land	Forest Land	Cropland	Change of BC(gha)
Z = 4					
Barren land	95.98	1.07	0.46	2.49	355009
Grazing land	0.00	76.89	18.22	4.89	216109
Forest Land	0.00	0.00	98.80	1.20	24538
Cropland	0.00	0.00	0.00	100	0
Change ratio	−4.01	−18.09	22.02	12.75	595656
Z = 8					
Barren land	95.62	1.22	0.44	2.72	387321
Grazing land	0.00	74.66	19.72	5.62	243017
Forest Land	0.00	0.00	98.76	1.24	25415
Cropland	0.00	0.00	0.00	100	0
Change ratio	−4.38	−19.64	23.60	14.11	655753

**Table 2:** Transformation matrix of four types’ land use according to the OB scenario (%).

Pre-\Post-opt	Barren land	Grazing land	Forest Land	Cropland	Change of BC(gha)
Z = 4					
Barren land	95.97	1.28	0.26	2.49	351592
Grazing land	0.00	81.46	13.68	4.86	195098
Forest Land	0.00	7.68	91.17	1.15	−5962
Cropland	0.00	3.20	1.93	94.87	−185105
Change ratio	−4.03	−1.37	11.20	7.58	355623
Z = 8					
Barren land	95.55	1.37	0.38	2.70	384505
Grazing land	0.00	79.64	14.87	5.49	218000
Forest Land	0.00	9.02	89.80	1.18	−10722
Cropland	0.00	3.50	2.13	94.37	−203134
Change ratio	−4.45	−1.20	12.17	8.29	388649

**Table 3:** Transformation matrix of four types’ land use according to the OBC scenario (%).

Pre-\Post-opt	Barren land	Grazing land	Forest Land	Cropland	Change of BC(gha)
Z = 4					
Barren land	98.41	1.25	0.34	0.00	15434
Grazing land	0.00	86.19	13.81	0.00	60815
Forest Land	0.00	7.80	92.20	0.00	−30119
Cropland	0.00	3.23	1.99	94.78	−188338
Change ratio	−1.59	3.37	12.91	−5.22	−142208
Z = 8					
Barren land	98.21	1.49	0.30	0.00	15746
Grazing land	0.00	85.51	14.49	0.00	63786
Forest Land	0.00	9.43	90.57	0.00	−36390
Cropland	0.00	3.35	2.29	94.36	−202664
Change ratio	−1.79	5.40	12.34	−5.64	−159522

**Table 4:** Transformation matrix of four types’ land use according to the OBW scenario (%).

Pre-\Post-opt	Barren land	Grazing land	Forest Land	Cropland	Change of BC(gha)
Z = 4					
Barren land	99.78	0.16	0.04	0.02	4160
Grazing land	0.00	98.07	1.89	0.04	9376
Forest Land	0.00	1.45	98.55	0.00	−5611
Cropland	0.00	3.32	1.63	95.05	−180152
Change ratio	−0.22	4.67	3.51	−4.87	−172258
Z = 8					
Barren land	99.76	0.16	0.06	0.02	5211
Grazing land	0.00	97.90	2.04	0.06	10557
Forest Land	0.00	1.54	98.42	0.04	−5065
Cropland	0.00	3.69	1.79	94.52	−199469
Change ratio	−0.24	5.10	3.91	−5.35	−188767

**Table 5:** Water resource demanded in the optimization of BC (Unit: 10^8^ m^3^).

Neighborhood	MB Scenario	OB Scenario	OBC Scenario
Z = 4	5.16	4.94	2.07
Z = 8	5.67	5.48	2.51

The water resource per square kilometers of different land use categories was provided by the yearbook.

**Table 6:** Change ratio of ecological overshoot in different scenarios (%).

Neighborhood	MB Scenario	OB Scenario	OBC Scenario	OBW Scenario
Z = 4	−47.87	−28.58	11.43	13.84
Z = 8	−52.70	−31.23	12.82	15.17

## References

[b1] Singh R. K., Murty H. R., Gupta S. K. & Dikshit A. K. An overview of sustainability assessment methodologies. Ecol Indic. 9(2), 189–212 (2009).

[b2] Carey D. I. Development based on carrying capacity: a strategy for environmental protection. Global Environ Chang. 3(2), 140–148 (1993).

[b3] Scoones I. Economic and ecological carrying capacity: applications to pastoral systems in Zimbabwe (ed. Barbier E. B.) Economics and Ecology: New Frontiers and Sustainable Development 96–117 (Chapman & Hall, London, 1993).

[b4] Hui C. Carrying capacity of the environment (ed. Wright J. D.) International Encyclopedia of the Social and Behavioral Sciences 155–160 (2nd Edition, Vol 3, Elsevier, Oxford, 2015).

[b5] Rees W. E. Ecological footprints and bio-capacity: essential elements in sustainability assessment (eds. Dewulf J., Van Langenhove H.) Renewables-Based Technology: Sustainability Assessment 143–158 (John Wiley and Sons, Chichester, 2006).

[b6] Lieth H. Modeling the primary productivity of the world. Nature and Resources. 8(2), 5–10 (1972).

[b7] Rees W. E. Ecological footprint and appropriated carrying capacity: what urban economics leaves out. Environ Urban. 4, 121–130 (1992).

[b8] Odum H. T. Environmental Accounting: Emergy and Environmental Decision Making (John Wiley and Sons, New York, 1996).

[b9] Kitzes J. . Shrink and share: humanity’s present and future ecological footprint. Phil Trans R Soc Lond B Biol Sci. 363(1491), 467–475 (2008). 17652075 10.1098/rstb.2007.2164 PMC2610164

[b10] Wackernagel M. & Yount J. D. Footprints for sustainability: the next steps. Environ Dev Sustain. 2, 21–42 (2000).

[b11] Senbel M., McDaniels T. & Dowlatabadi H. The ecological footprint: a non-monetary metric of human consumption applied to North America. Global Environ Chang. 13(2), 83–100 (2003).

[b12] Yue D. . Spatiotemporal analysis of ecological footprint and biological capacity of Gansu, China 1991–2015: down from the environmental cliff. Ecol Econ. 58, 393–406 (2006).

[b13] Yue D. . Biocapacity supply and demand in Northwestern China: a spatial appraisal of sustainability. Ecol Econ. 70, 988–994 (2011).

[b14] Bagliani M., Gallic A., Niccoluccic V. & Marchettini N. Ecological footprint analysis applied to a sub-national area: the case of the Province of Siena (Italy). J Environ Manage. 86, 354–364 (2008). 17110019 10.1016/j.jenvman.2006.04.015

[b15] Schoemburg J. Africa faces rising ecological deficit. Front Ecol Environ. 6(6), 295 (2008).

[b16] Venetoulis J. & Talberth J. Refining the ecological footprint. Environ Dev Sustain. 10(4), 441–469 (2008).

[b17] Kitzes J., Moran D., Galli A., Wada Y. & Wackernagel M. Interpretation and application of the ecological footprint: a reply to Fiala (2008). Ecol Econ. 68, 929–930 (2009).

[b18] Bastianoni S., Niccolucci V., Pulselli R. M. & Marchettini N. Indicator and indicandum: “Sustainable way” vs “prevailing conditions” in the Ecological Footprint. Ecol Indic. 16, 47–50 (2012).

[b19] Niccolucci V., Tiezzi E., Pulselli F. M. & Capineri C. Biocapacity vs Ecological Footprint of world regions: A geopolitical interpretation. Ecol Indic. 16, 23–30 (2012).

[b20] Holden E., Linnerud K. & Banister D. Sustainable development: our common future revisited. Global Environ Chang. 26, 130–139 (2014).

[b21] Rugani B., Roviani D., Hild P., Schmitt B. & Benetto E. Ecological deficit and use of natural capital in Luxembourg from 1995 to 2009. Sci Total Environ. 468, 292–301 (2014). 24036220 10.1016/j.scitotenv.2013.07.122

[b22] Galli A. On the rationale and policy usefulness of Ecological Footprint Accounting: The case of Morocco. Environ Sci Policy. 48, 210–224 (2015).

[b23] WWF. Living Planet Report 2010 (WWF International, Gland, Switzerland, 2010).

[b24] WWF. Living Planet Report 2012 (WWF International, Gland, Switzerland, 2012).

[b25] WWF. Living Planet Report 2014: species and spaces, people and places (WWF International, Gland, Switzerland, 2014).

[b26] Rees W. E. & Wackernagel M. Ecological footprints and appropriated carrying capacity: measuring the natural capital requirements of the human economy. (eds Jansson A. M., Hammer M., Folke C. & Costanza R.) 362–390 (Investing in Natural Capita 1: The Ecological Economics Approach to Sustainability. Island Press, Washington DC, 1994).

[b27] Yue D., Guo J. J. & Hui C. Scale dependency of biocapacity and the fallacy of unsustainable development. J Environ Manage. 126, 13–19 (2013). 23648317 10.1016/j.jenvman.2013.04.022

[b28] Haberl H., Erb K. H. & Krausmann F. How to calculate and interpret ecological footprint for long periods of time: the case of Austria 1926–1995. Ecol Econ. 38, 25–45 (2001).

[b29] Zhao S., Li Z. & Li W. A modified method of ecological footprint calculation and its application. Ecol Model. 185(1), 65–75 (2005).

[b30] Siche R., Agostinho F. & Ortega E. Emergy net primary production (ENPP) as basis for calculation of ecological footprint. Ecol Indic. 10(2), 475–483 (2010).

[b31] Bond W. J. Ancient grasslands at risk. Science. 351(6269), 120–122 (2016). 26744392 10.1126/science.aad5132

[b32] Van den Bergh, Jeroen C. J. M. & Fabio G. Ecological footprint policy? Land use as an environmental indicator. J Ind Ecol. 18(1), 10–19 (2014).

[b33] Wackernagel M. Comment on “ecological footprint policy? land use as an environmental indicator”. J Ind Ecol. 18(14), 20–23 (2014).

[b34] Ferng J. J. Toward a scenario analysis framework for energy footprints. Ecol Econ. 40(1), 53–69 (2002).

[b35] Van den Bergh, Jeroen C. J. M. & Fabio G. Response to Wackernagel. J Ind Ecol. 18(1), 23–25 (2014).

[b36] Chang B. & Xiong L. Ecological footprint analysis based on RS and GIS in arid land. J Geogr Sci. 15, 44–52 (2005).

[b37] Guo J. J. . Effects of the spatial scale on regional biocapacity-a case study of Shiyang River Basin. Journal of Lanzhou University (Natural Sciences Edition). 50(3), 383–389 (In Chinese) (2014).

[b38] Rounsevell M. D. A. & Metzger M. J. Developing qualitative scenario storylines for environmental change assessment. WIRES Clim Change. 1, 606–619 (2010).

[b39] Mancosu E. . Future land-use change scenarios for the Black Sea catchment. Environ Sci Policy. 46, 26–36 (2015).

[b40] Leimbach M. . Future growth patterns of world regions–A GDP scenario approach. Global Environ Chang. http://dx.doi.org/10.1016/j.gloenvcha.2015.02.005 (2015).

[b41] Wood G. Modelling the ecological footprint of green travel plans using GIS and network analysis: from metaphor to management tool? Environ Plann B. 30, 523–540 (2003).

[b42] Roura-Pascual N., Krug R. M., Richardson D. M. & Hui C. Spatially-explicit sensitivity analysis for conservation management: exploring the influence of decisions in invasive alien plant management. Divers Distrib. 16(3), 426–438 (2010).

[b43] Aguiar A. P. D. . Land use change emission scenarios: Anticipating a forest transition process in the Brazilian Amazon. Global change biol. 22, 1821–1840 (2016). 10.1111/gcb.13134 26511401

[b44] Lin D., Wackernagel M., Galli A. & Kelly R. Ecological Footprint: Informative and evolving–A response to van den Bergh and Grazi (2014). Ecol Indic. 58, 464–468 (2015).

[b45] Bryan B. A. . Land-use and sustainability under intersecting global change and domestic policy scenarios: trajectories for Australia to 2050. Global Environ Chang. 38, 130–152 (2016).

[b46] Tilman D., Cassman K. G., Matson P. A., Naylor R. & Polasky S. Agricultural sustainability and intensive production practices. Nature 418(6898), 671–677 (2002). 12167873 10.1038/nature01014

[b47] Scherer L. & Pfister S. Dealing with uncertainty in water scarcity footprints. Environ Res Lett. 11(5), 054008 (2016).

[b48] Wackernagel M. Tracking the ecological overshoot of the human economy. P Natl Acad Sci USA 99(14), 9266–9271 (2002). 10.1073/pnas.142033699 PMC123129 12089326

[b49] Hui C. On the scaling patterns of species spatial distribution and association. J Theor Biol. 261(3), 481–487 (2009). 19699752 10.1016/j.jtbi.2009.08.015

[b50] Han X. & Hui C. Niche Construction on Environmental Gradients: The Formation of Fitness Valley and Stratified Genotypic Distributions. PLOS ONE. e99775 (2014). 10.1371/journal.pone.0099775 PMC4051751 24915290

[b51] Shao J. A., Li Y. B., Wei C. F. & Xie D. The drivers of land use change at regional scale: assessment and prospects. Adv Earth Sci. 22(8), 798–809 (In Chinese) (2007).

[b52] Gao P., Niu X., Wang B. & Zheng Y. L. Land use changes and its driving forces in hilly ecological restoration area based on gis and rs of northern china. Sci Rep. 5, 11038, doi: 10.1038/srep11038 (2015). 26047160 PMC4457013

[b53] Kremen C. . Economic incentives for rain forest conservation across scales. Science. 288(5472), 1828–1832 (2000). 10846165 10.1126/science.288.5472.1828

[b54] Liming H. Financing rural renewable energy: a comparison between China and India. Renew Sust Energ Rev. 13(5), 1096–1103 (2009).

[b55] Bryan B. A. & Crossman N. D. Impact of multiple interacting financial incentives on land use change and the supply of ecosystem services. Ecosyst Serv. 4, 60–72 (2013).

[b56] Reeson A. 2008. Institutions, motivations and public goods: theory, evidence and implications for environmental policy. Socio-Economics and the Environment in Discussion. CSIRO Working Paper Series 2008-01. CSIRO Sustainable Ecosystems, Canberra (2008).

[b57] Jiang B. H. Driving Force to Biocapacity of Multi-scale Areas in Shiyang River Basin. Lanzhou University (Master dissertation, 2016).

[b58] Wang Y. Q. . Spatial-temporal dynamic analysis of the biocapacity pattern in the mountain area of upper Shiyang River catchment. Journal of Lanzhou University (Natural Sciences Edition). 49(2), 166–172 (In Chinese) (2014).

[b59] Jiang B. H. . Spatio-Temporal transfer changes of biocapacity in oasis areas of the middle and lower Shiyang River Catchment. Journal of Lanzhou University (Natural Sciences Edition). 51(5), 625–632 (2015).

[b60] Li Q. & Ren, Z. Y. Driving forces and dynamic simulation of agricultural land in the southern Loess Plateau. J Arid Land Resour Environ. 26(11), 1–5 (2012).

[b61] Braimoh A. K. & Onishi T. Spatial determinants of urban land use change in Lagos, Nigeria. Land Use Policy. 24(2), 502–515 (2007).

[b62] Huang J., Gao J. & Zhang Y. Eutrophication Prediction Using a Markov Chain Model: Application to Lakes in the Yangtze River Basin, China. Environ Model Assess. 21(2), 233–246 (2016).

[b63] Tang H. J., Chen Y. J., Qiu J. J. & Chen Z. X. China’s land use/land cover change research. China Agriculture Press (2004).

[b64] Omar N. Q., Ahamad M. S. S., Hussin W. M. A. W., Samat N. & Ahmad S. Z. B. Markov CA, multi regression, and multiple decision making for modeling historical changes in Kirkuk City, Iraq. J Indian Soc Remote. 42(1), 165–178 (2014).

[b65] Guo J. J. Spatial Scale Effect and Optimization of Basin’s Biocapacity-Case Studies of Shiyang River Basin and Jinghe River Watershed. Lanzhou University (Doctoral dissertation, 2014).

[b66] Turner M. G. Spatial simulation of landscape changes in Georgia: a comparison of 3 transition models. Landscape Ecol. 1(1), 29–36 (1987).

[b67] Barredo J. I., Kasanko M., McCormick N. & Lavalle C. Modelling dynamic spatial processes: simulation of urban future scenarios through cellular automata. Landscape Urban Plan. 64(3), 145–160 (2003).

[b68] White R. & Engelen G. Urban systems dynamics and cellular automata: fractal structures between order and chaos. Chaos Soliton Fract. 4(4), 563–583 (1994).

[b69] Schneider L. C. & Pontius R. G. Modeling land-use change in the Ipswich watershed, Massachusetts, USA. Agr Ecosyst Environ. 85(1), 83–94 (2001).

[b70] Jiang F. C. . Features of space distribution of the forest line and relations between the forest line and climatic limit of permafrost and climatic snowline in China. J Geomech. 10(4), 289–299 (2004).

