# Supplementary material for: Biocapacity optimization in regional planning
Source: Sci Rep. 2017 Jan 23;7:41150. doi: 10.1038/srep41150 (PMC5253737; doi:10.1038/srep41150)
Supplement: Supplementary Information [file srep41150-s1.pdf]

Supplementary Information

Biocapacity optimization in regional planning

Jianjun Guo[1,2,†], Dongxia Yue[1, 2,*, †], Kai Li[1], Cang Hui[3,4]

[1]Key Laboratory of Western China's Environmental Systems（Ministry of Education), College of Earth and Environmental Sciences, Lanzhou University, Lanzhou, 730000, China

[2]Northwest Institute of Eco-Environment and Resources, Chinese Academy of Sciences, Lanzhou 730000, China

[3]Centre for Invasion Biology, Department of Mathematical Sciences, Stellenbosch University, Matieland 7602, South Africa

[4]Mathematical Biosciences Group, African Institute for Mathematical Sciences, Cape Town 7945, South Africa

Content:

Figure. S1 Dynamic map of the sizes of four types' land use according to the MB scenario.
Figure. S2 Dynamic map of the sizes of four types' land use according to the OB scenario.
Figure. S3 Dynamic map of the sizes of four types' land use according to the OBC scenario.
Figure. S4 Dynamic map of the sizes of four types' land use according to the OBW scenario.

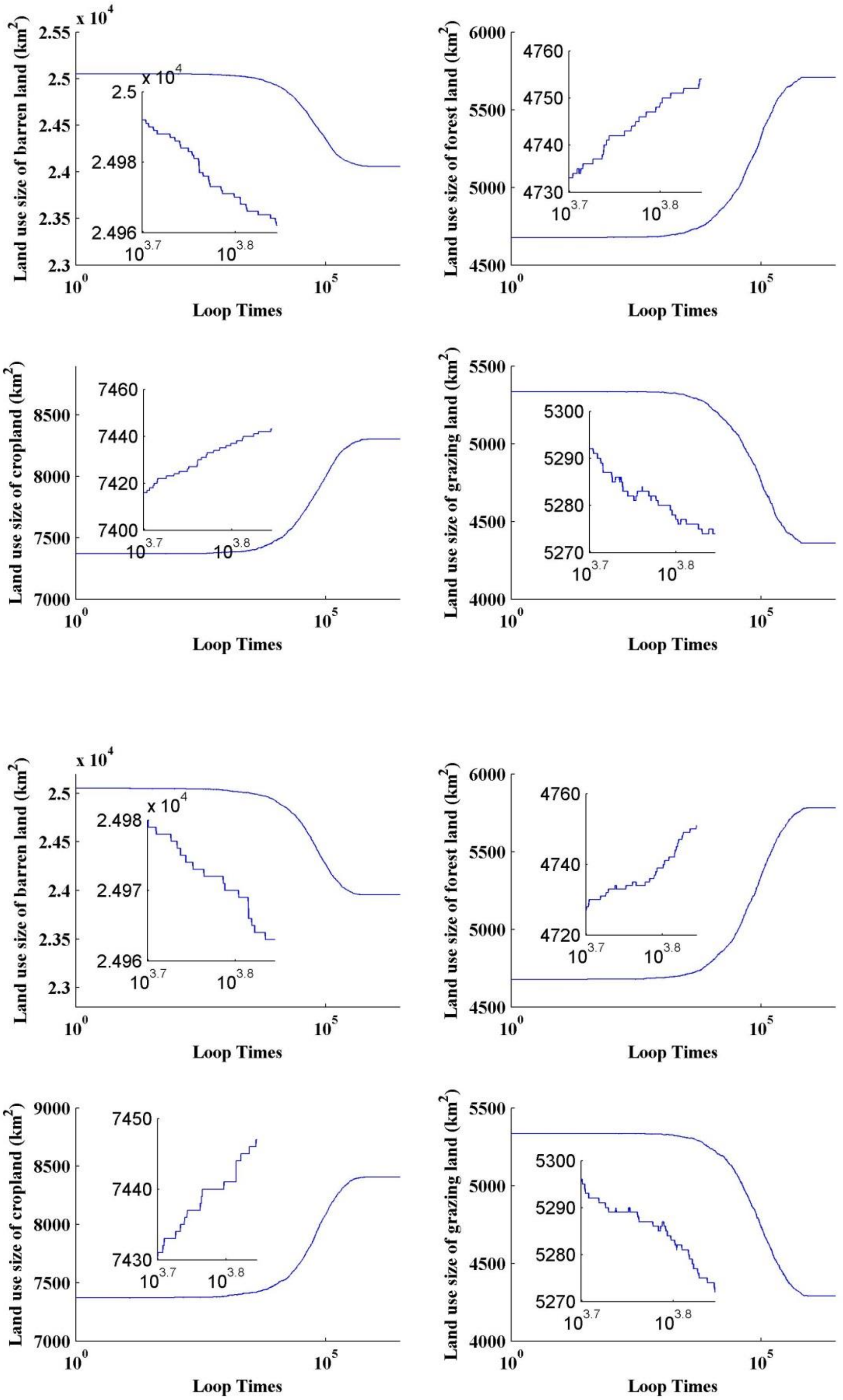

Figure S1. Dynamic map of the sizes of four types' land use according to the MB scenario (top: Z=4; bottom: Z=8), the nested small figure stands for the dynamic map when the loop times is of 5000-7000.

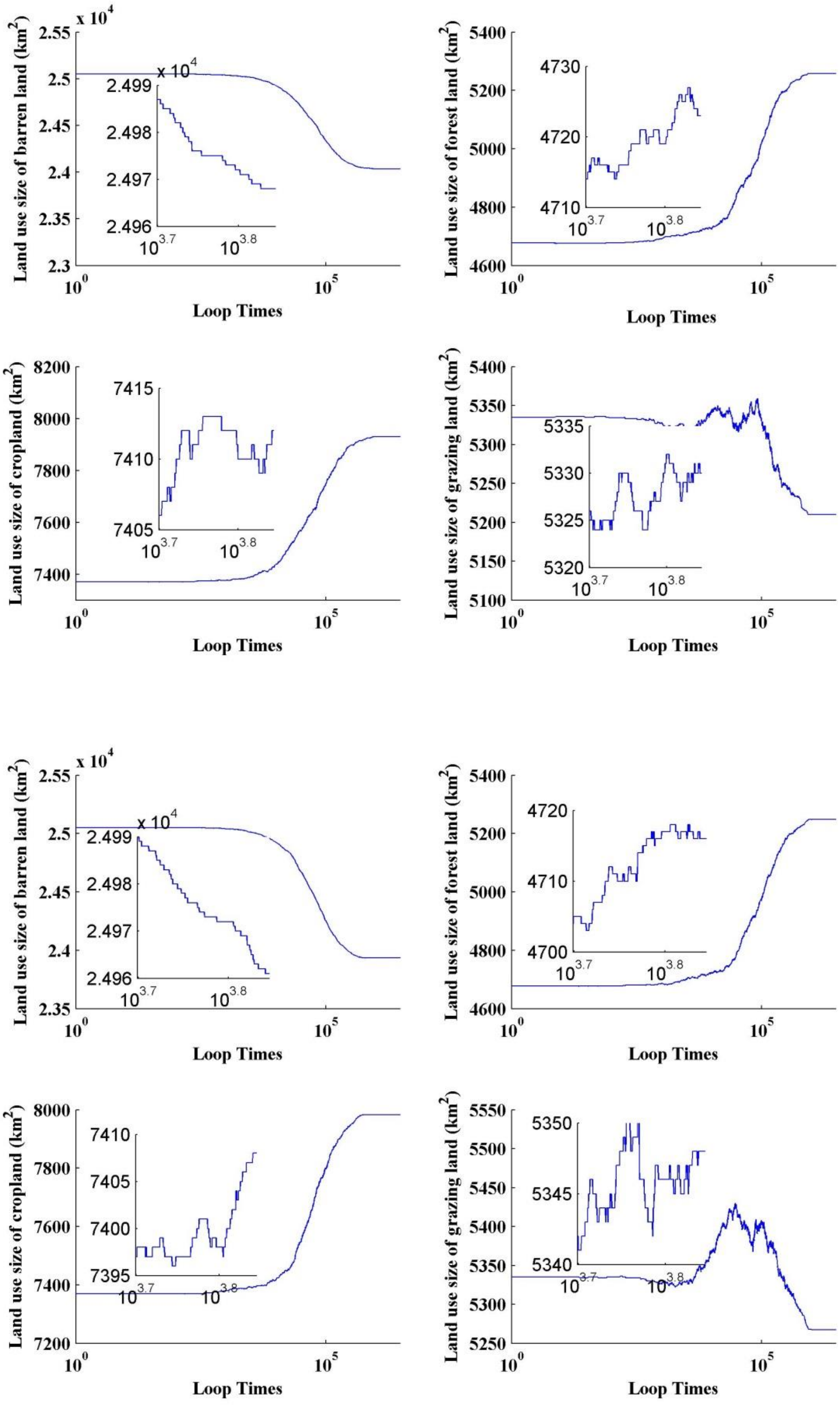

Figure S2. Dynamic map of the sizes of four types' land use according to the OB scenario (top: Z=4; bottom: Z=8), the nested small figure stands for the dynamic map when the loop times is of 5000-7000.

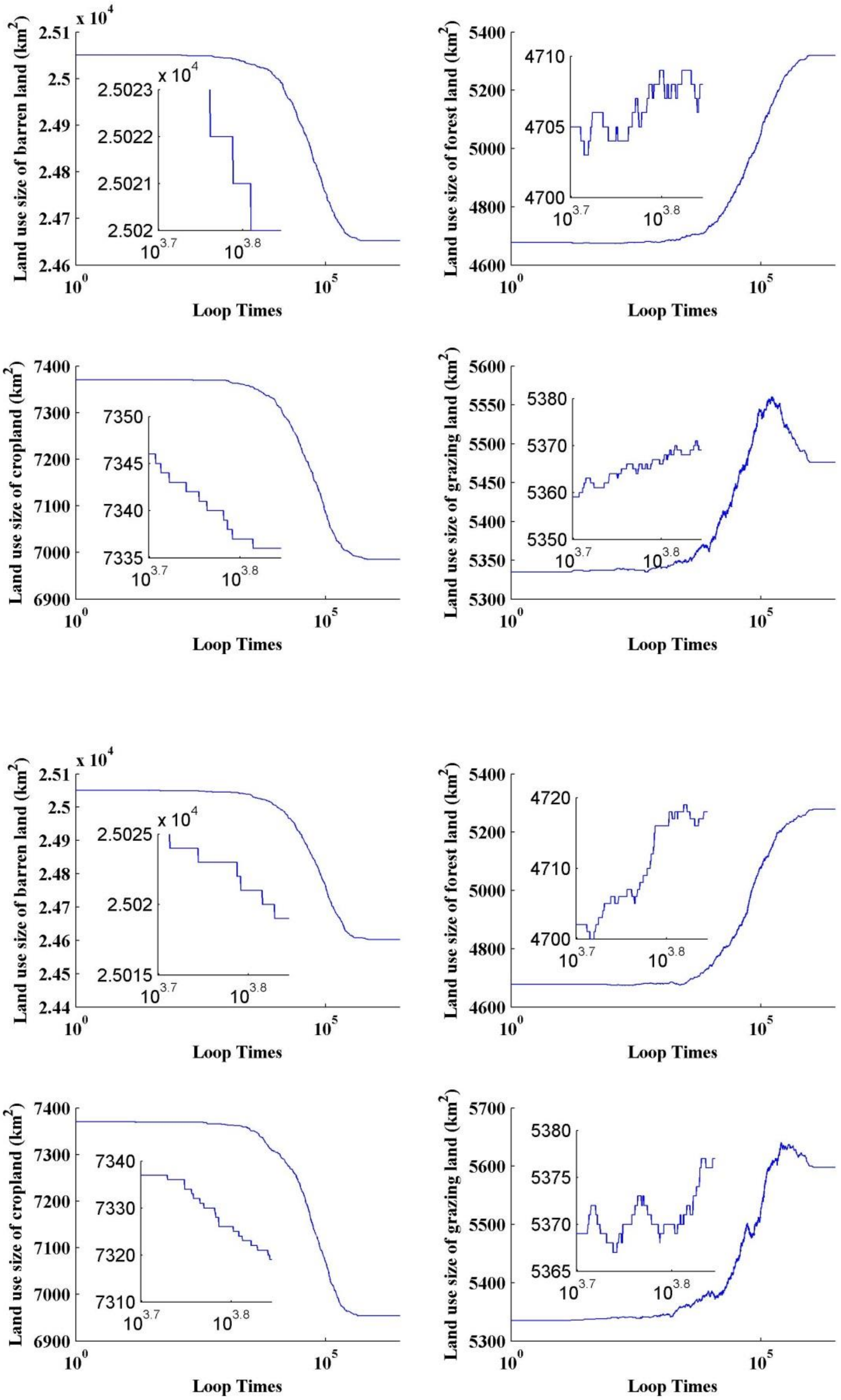

Figure S3. Dynamic map of the sizes of four types' land use according to the OBC scenario (top: Z=4; bottom: Z=8), the nested small figure stands for the dynamic map when the loop times is of 5000-7000.

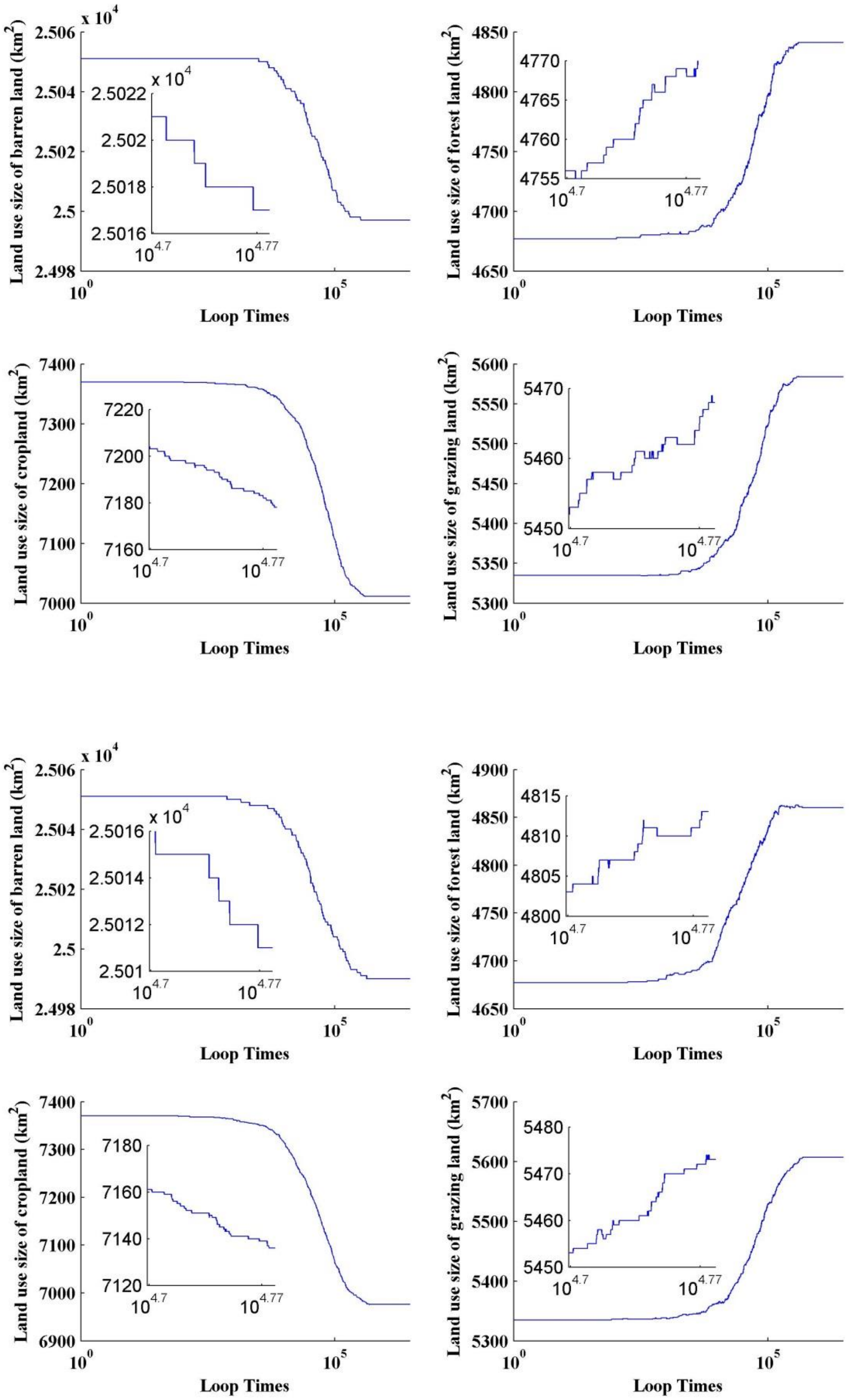

Figure S4. Dynamic map of the sizes of four types' land use according to the OBW scenario (top: Z=4; bottom: Z=8), the nested small figure stands for the dynamic map when the loop times is of 5000-7000.
